# Public Awareness of Genetic Diseases, Preventive Screening, and Genetic Healthcare Services in the Qassim Region, Saudi Arabia: A Cross-Sectional Study

**DOI:** 10.3390/healthcare14162549

**Published:** 2026-08-14

**Authors:** Hitham Aldharee, Ghady Almutari, Alanoud Alofi, Mayyaz Alqubays, Hamdan Z. Hamdan

**Affiliations:** 1 Department of Pathology, College of Medicine, Qassim University, Buraidah 52571, Saudi Arabia; 2 Department of Diabetes Center, Medical City, Qassim University, Buraidah 52571, Saudi Arabia; 3 Department of Family Medicine, Security Force Hospital, Riyadh 11481, Saudi Arabia; 4 Department of Emergency, King Fahad Specialist Hospital, Buraidah 52366, Saudi Arabia; 5 Department of Obstetrics and Gynecology, Maternity and Children Hospital, Buraidah 52384, Saudi Arabia

**Keywords:** inherited diseases, genetics, public awareness, Qassim, Saudi Arabia

## Abstract

**Background:** Genetic diseases pose a significant public health issue in Saudi Arabia, driven by relatively high prevalence and factors such as consanguineous marriage. Increasing public awareness and understanding are crucial to improving prevention, early detection, and the use of genetic healthcare services. This study aimed to evaluate residents’ knowledge, attitudes, and awareness of genetic diseases in the Qassim region. **Methods:** A web-based cross-sectional survey was conducted from March to May 2024 among Qassim residents, using an online questionnaire shared via multiple social media platforms. The survey assessed knowledge of genetic diseases, awareness of preventive measures and screening programs, and familiarity with genetic healthcare services. Descriptive statistics summarised the results, and binary logistic regression was conducted to identify factors independently associated with good awareness of genetic testing. **Results:** Of the 398 participants, about 66% knew about common genetic diseases in Saudi Arabia, while 92% recognised consanguineous marriage as a key cause of genetic disorders. Most respondents correctly identified that genetic diseases are not transmitted through infection (93%). Support for expanding premarital, prenatal, and newborn screening programs was high at 93%, 88.9%, and 90.7%, respectively. Nonetheless, only 38.7% were aware of specialised genetic laboratories and counselling services, and just 4.4% had consulted a genetic counsellor. Social media was the primary source of genetic information (36%). Despite limited awareness of available services, 94% supported establishing a regional genetic testing lab, and 71% were willing to participate in future genetic research. Multivariate analysis identified that marital status is the only variable significantly associated with genetics testing awareness status [aOR = 2.34; 95% CI (1.08–5.08); *p* = 0.030]. **Conclusions:** Participants demonstrated generally good knowledge of genetic diseases and positive attitudes towards preventive genetic screening programmes. However, awareness of and utilisation of specialised genetic healthcare services, including genetic counselling and genetic testing laboratories, remained limited. These findings underscore the need to enhance public education, expand genetic counselling, and improve access to specialised services to advance genomic medicine in Saudi Arabia. However, the predominance of younger participants and students may limit the generalisability of the findings to the broader Qassim population.

## 1. Introduction

Genetic diseases result from harmful changes in genes, chromosomes, or the entire genome that disrupt normal biological processes and can cause a wide range of symptoms [1]. These diseases are generally grouped into three main types: single-gene (monogenic) disorders, chromosomal abnormalities, and multifactorial disorders arising from complex interactions between genes and environmental influences [1]. They can be inherited from one or both parents or arise spontaneously during a person’s life. Clinical symptoms vary widely, with some presenting at birth and others appearing later in life [1].

Genetic disorders worldwide pose a significant public health concern, affecting morbidity, mortality, and healthcare costs [1,2]. Common conditions include Down syndrome, cystic fibrosis, sickle cell disease, familial hypercholesterolaemia, and Turner syndrome. In Saudi Arabia, these disorders are particularly prevalent due to demographic and social factors, including high rates of consanguineous marriage, large families, and the persistence of inherited conditions in specific populations [3,4,5,6]. Notably, the Kingdom frequently reports cases of Down syndrome, cystic fibrosis, sickle cell disease, hereditary hearing loss, hereditary blindness, and congenital heart issues [4,5,6].

Innovations in genetic medicine have greatly enhanced the diagnosis, treatment, and prevention of hereditary diseases. Increased access to genetic testing, counselling, and nationwide screening programs has created better opportunities for early detection and intervention [7,8,9]. In Saudi Arabia, extensive efforts aim to reduce the impact of genetic disorders through public health strategies such as premarital screening, newborn and prenatal diagnostic services, and awareness campaigns. At the same time, the country is expanding its investment in genomic medicine and precision healthcare through efforts like the Saudi Human Genome Program. These initiatives aim to understand disease genetics and incorporate genomic data into prevention, diagnosis, and personalised treatment. Turning these efforts into tangible health benefits for the population requires more than just technological and healthcare infrastructure; it also depends on improving public understanding of genetics, increasing awareness of genetic testing and screening, and familiarizing the population with available genetic healthcare services.

Public knowledge and awareness are crucial for identifying misconceptions, assessing the effectiveness of current educational efforts, and informing future health promotion strategies. These measurements are closely linked to genetic literacy, which generally reflects a person’s capacity to understand and use genetic information for health decisions. Enhancing genetic literacy is especially vital as genetic testing, screening, and genomic medicine become more integrated into healthcare. Although genetic literacy is vital, limited data exist on public awareness of genetic diseases in the Qassim region. This study aimed to evaluate residents’ knowledge, attitudes, and awareness of genetic diseases in the Qassim region of Saudi Arabia. Specifically, it assesses public understanding and awareness, knowledge of preventive measures and screening programs, and familiarity with available healthcare and genetic services for those affected by genetic conditions.

## 2. Methods

### 2.1. Study Design

This study used a web-based cross-sectional survey to evaluate residents of the Qassim region in Saudi Arabia on their knowledge, awareness, and attitudes toward genetic diseases, preventive genetic screening, and genetic healthcare services. Data were gathered from March to May 2024 through a structured, self-administered online questionnaire.

### 2.2. Study Population

The study targeted residents of the Qassim region in Saudi Arabia. Participants were recruited via an online questionnaire distributed on social media platforms, including WhatsApp (version 26.12.78) and X (formerly Twitter, version v10.83.0). Recruitment was not restricted to any particular educational or occupational group. Participation was voluntary, and participants had to reside in the Qassim region.

### 2.3. Eligibility Criteria

Saudi residents of the Qassim region who could read and understand Arabic and who voluntarily agreed to participate in the online questionnaire were eligible for inclusion in the study. Non-Saudis, as well as those with substantial missing data or incomplete responses, were excluded from the final analysis. All participants provided electronic informed consent before completing the questionnaire.

### 2.4. Questionnaire

The questionnaire, adapted from a prior study [10] and tailored to this study’s objectives, was translated into Arabic and reviewed by experts in medical genetics and public health to assess its content validity, clarity, and cultural appropriateness before distribution. Formal pilot testing and reliability assessment were not performed. The questionnaire was divided into four sections: demographic details (five questions), knowledge and awareness of genetic diseases (thirteen questions), understanding of genetic diagnosis and prevention (seven questions), and sources of information about genetic diseases (three questions). The complete questionnaire is provided as Supplementary Material S1.

### 2.5. Sample Size

The minimum sample size was calculated using the Epi Info statistical calculator (version 7.2). Based on an estimated population of 1 million in the Qassim region, with a 95% confidence level and a 5% margin of error, the required sample size was 384 participants. Ultimately, 398 responses were collected and included in the final analysis.

### 2.6. Ethical Approval

This study received ethical approval from the Institutional Review Board (IRB) of Qassim University in Saudi Arabia (Approval No. 24-84-09). Participation was voluntary, and informed consent was obtained electronically before participants completed the questionnaire.

### 2.7. Data Analysis

The data were exported to Microsoft Excel for review to ensure completeness and accuracy before analysis. Responses with missing or incomplete details were examined and excluded. Statistical Package for the Social Sciences (SPSS) for Windows (version 16.0) was used to analyse the data. Descriptive statistics summarised participants’ characteristics and survey responses, with results presented as frequencies and percentages. The chi-square test was used to compare categorical variables. Univariate and multivariate regression analyses were conducted to identify variables associated with genetic testing awareness status (high awareness vs. moderate and low awareness). To determine participants’ level of awareness, a composite awareness score was constructed using the knowledge-based items included in the questionnaire (7 items). The seven items were selected because they assessed participants’ knowledge and awareness of genetic diseases and were suitable for inclusion in the composite awareness measure. Items assessing attitudes, perceptions, healthcare services, and willingness to engage in genetic research were excluded from the composite score. Each item was scored as 1 for a correct response and 0 for an incorrect response, with 1 negatively worded question reverse-coded. The individual item scores were then summed to obtain a total awareness score ranging from 0 to 7. Participants were classified into three awareness categories according to the percentage of correct responses as described previously [11]. Participants who answered 80% or more of the knowledge questions correctly (6–7 correct responses) were classified as having good awareness; those with 60–79% correct responses (4–5 correct responses) were classified as having moderate awareness; and those with less than 60% correct responses (0–3 correct responses) were classified as having poor awareness. Seven questions were used to assess awareness. Awareness status is considered the dependent variable, and variables including participants’ age, sex, marital status, occupation, residence, family history of hereditary diseases, and educational level were considered independent variables. Factors whose *p*-values reach or exceed 0.200 are qualified to be considered in the multivariate analysis. Crude odds ratios (ORs) and adjusted odds ratios (aORs), along with the 95% confidence intervals, are reported. A probability value of less than 0.05 was determined as a cutoff to reject the null hypothesis. For regression analyses, the awareness score was dichotomized by classifying participants with good awareness as the reference category for comparison with those with poor or moderate awareness.

### 2.8. Variable Definitions

In this study, the constructs were defined as follows: Knowledge pertains to participants’ factual understanding of genetic diseases, including causes, inheritance, diagnosis, prevention, and genetic testing options. Awareness relates to their familiarity with genetic healthcare services, preventive screening programs, genetic counselling, and the availability of specialized genetic services within the healthcare system. Attitudes encompass participants’ beliefs, perceptions, acceptance, and willingness to engage in preventive genetic screening, genetic counselling, and other genetics-related healthcare activities.

## 3. Results

### 3.1. Sociodemographic Characteristics of Participants

A total of 398 individuals participated in the survey. Most respondents were female (71.9%, n = 286), while males made up 28.1% (n = 112). Nearly half of participants (49.5%, n = 197) were aged 21–25, followed by those over 36 (17.8%, n = 71) and those aged 16–20 (16.6%, n = 66). Significant differences were observed between males and females across age groups (*p* < 0.001) (Table 1). Most participants held a bachelor’s degree (67.8%, n = 270), while 19.8% (n = 79) had completed secondary school. There was a significant difference in educational level between males and females (*p* = 0.001). Most respondents were single (73.4%, n = 292), with 24.1% (n = 96) married. No significant gender difference was observed in marital status (*p* = 0.168).

Students were the largest occupational group at 82.4% (n = 328), followed by individuals in other occupations at 14.1% (n = 56). There were notable differences in occupational status between males and females (*p* < 0.001). Participants were drawn from various cities in the Qassim region, primarily Buraidah (31.9%, n = 127) and Unaizah (24.5%, n = 98). However, no significant gender difference was observed in place of residence (*p* = 0.960) (Table 1).

### 3.2. Knowledge and Awareness of Genetic Diseases

Overall, 66.3% (n = 264) of participants reported knowing the most common genetic diseases in Saudi Arabia, whereas 33.7% (n = 134) reported no such knowledge. Nearly half of respondents (46.0%, n = 183) considered genetic diseases to be very common in the Qassim region, while 35.7% (n = 142) were uncertain. A large majority of participants (92.5%, n = 368) believed that consanguineous marriage is one of the major causes of genetic diseases. Furthermore, 93.7% (n = 373) correctly indicated that genetic diseases are not transmitted through infection (Table 2).

Regarding disease prevention, 61.3% (n = 244) reported knowing how to reduce the risk of developing genetic diseases, while 51.5% (n = 205) reported knowing how genetic diseases are diagnosed. In addition, 85.4% (n = 340) believed that individuals diagnosed with genetic diseases can have healthy children. No statistically significant differences were observed between males and females for any of the knowledge-related questions (*p* > 0.05) (Table 2).

**Figure 1:**
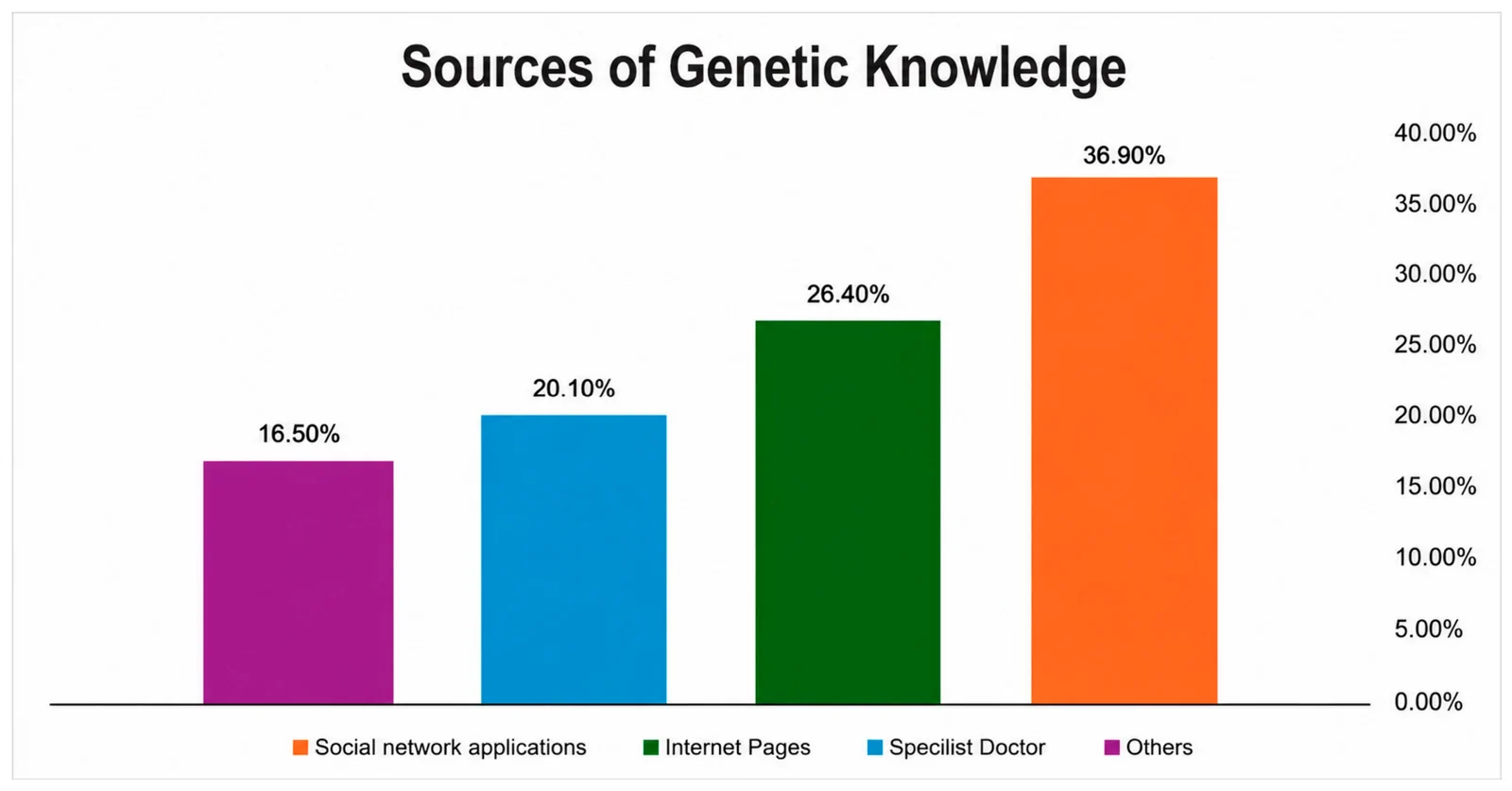
Sources of genetic knowledge reported by participants from the Qassim region, Saudi Arabia. Percentages indicate the proportion of respondents citing each source of genetic information.

Regarding sources of genetic knowledge, social media platforms represented the most common source of information (36.9%), followed by internet websites (26.4%), specialist physicians (20.1%), and other sources (16.5%) (Figure 1).

**Figure 2:**
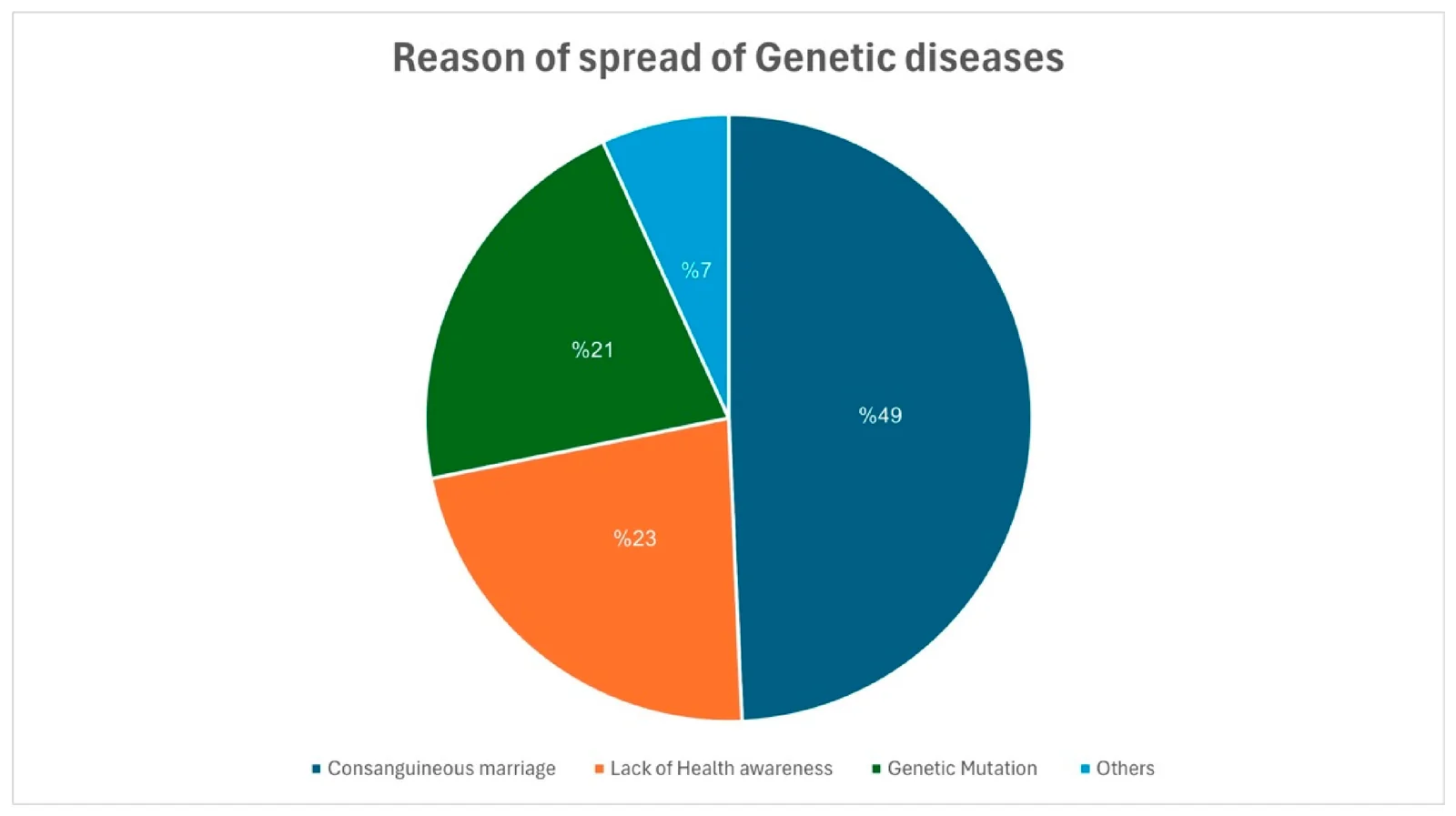
This figure shows participants’ perceptions of factors contributing to the spread of genetic diseases in the Qassim region, Saudi Arabia. Respondents could choose multiple options; the percentages indicate how many selected each factor.

Participants identified several causes for the spread of genetic diseases in the Qassim region. Consanguineous marriage was considered the leading factor by 49% of respondents, followed by lack of health awareness (23%), genetic mutations (21%), and other causes (7%) (Figure 2).

### 3.3. Attitudes Toward the Prevention of Genetic Diseases

Approximately 23.1% (n = 92) of participants reported a family member affected by a hereditary disease. Over half (58.8%, n = 234) knew where to find information if a genetic disease was present in their family (Table 3).

Similarly, 56.8% (n = 226) reported knowing what actions to take if they discovered they could pass on a genetic disease to their children. There was strong support for expanding genetic screening programs. Specifically, 93.5% (n = 372) supported increasing the number of conditions included in premarital screening, 88.9% (n = 354) supported expanding prenatal screening, and 90.7% (n = 361) supported increasing the number of disorders screened for in newborn screening programs. Significant gender differences were observed in support for expanding premarital screening (*p* = 0.035), prenatal screening (*p* = 0.046), and newborn screening (*p* = 0.011), with females generally expressing greater support than males (Table 3).

**Figure 3:**
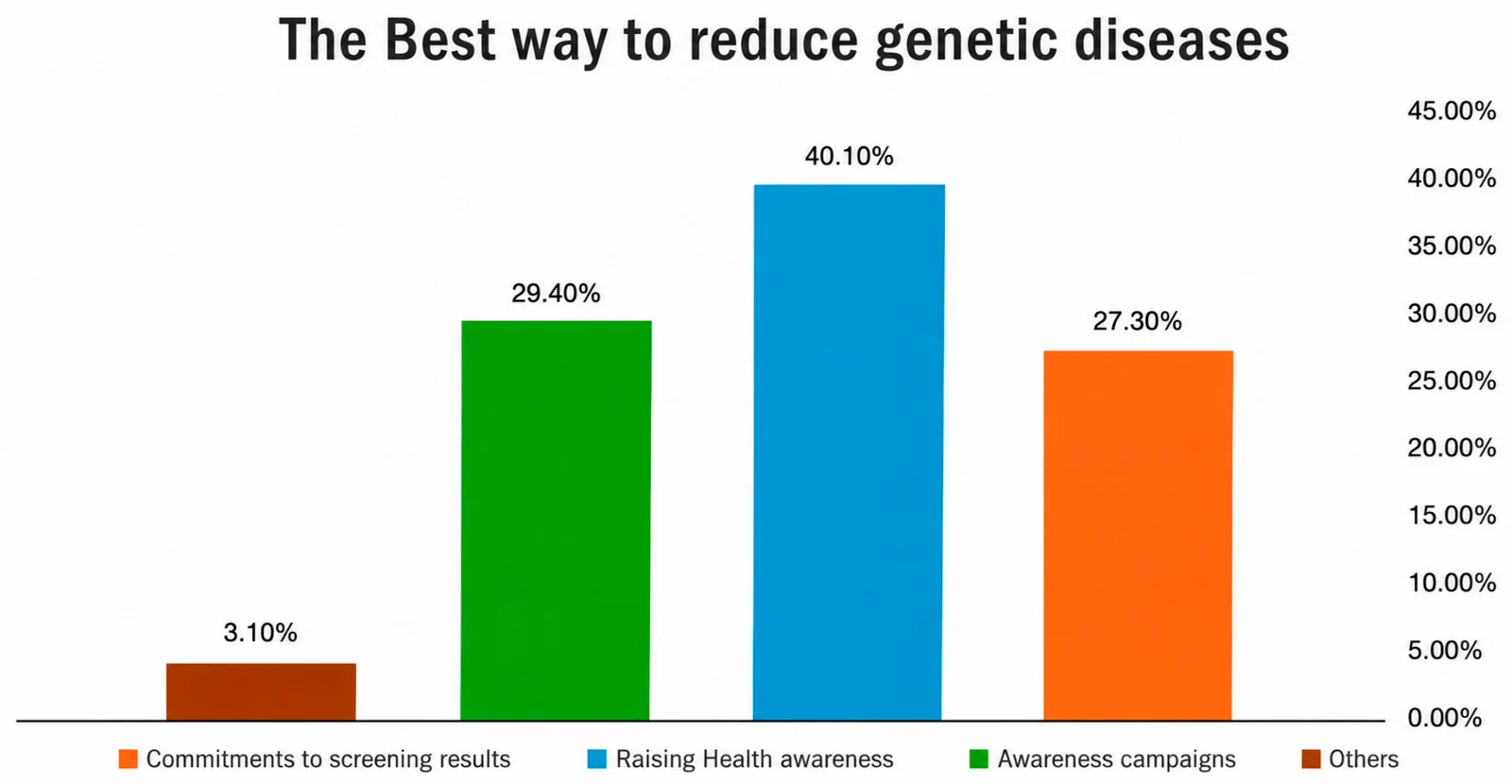
This figure shows participants’ opinions on the most effective ways to reduce the impact of genetic diseases in the Qassim region, Saudi Arabia. Multiple options could be chosen, and the percentages reflect the share of respondents who selected each method.

When asked about the most effective approach to reducing genetic diseases in the Qassim region, participants most frequently selected raising health awareness (40.1%), followed by awareness campaigns (29.4%), commitment to screening results (27.3%), and other approaches (3.1%) (Figure 3).

### 3.4. Awareness of Genetic Health Services in the Qassim Region

Slightly more than half of the participants (51.8%, n = 206) believed that healthcare services for individuals with hereditary diseases have improved in the Qassim region, whereas 48.2% (n = 192) did not share this view. This perception differed significantly between males and females (*p* = 0.045) (Table 4). Only 38.7% (n = 154) of the respondents reported being aware of specialised genetic testing laboratories and genetic counselling clinics in their cities. Despite this, 94.0% (n = 374) supported establishing a regional laboratory dedicated to genetic testing services.

Very few participants had previously contacted a genetic counsellor (4.5%, n = 18) or participated in research studies on genetic diseases (10.6%, n = 42). Nevertheless, 71.6% (n = 285) expressed willingness to participate in future studies on genetic diseases (Table 4).

Univariate regression analysis showed that only marital status [OR = 1.73; 95% CI (1.05–2.85); *p* = 0.029] and occupation [OR = 0.69; 95% CI (0.51–0.94); *p* = 0.019] were significantly associated with genetic testing awareness status (Table 5). Among the variables entered into the multivariate analysis model, only marital status was found to be significantly associated with genetic testing awareness status [aOR = 2.34; 95% CI (1.08–5.08); *p* = 0.030] (Table 5).

## 4. Discussion

This study evaluated how residents of the Qassim region in Saudi Arabia understand, perceive, and are aware of genetic diseases. Results indicated generally good awareness of genetic conditions and their risk factors, particularly the significance of consanguineous marriages. A key finding of this study was the high awareness of the link between consanguineous marriage and genetic diseases. Over 90% of participants acknowledged consanguinity as a major factor, with nearly half citing it as the primary cause of genetic conditions in the Qassim region. This is significant in Saudi Arabia, where consanguineous marriages remain common [3]. Past research shows consanguinity rates above 50% in Saudi communities, which contributes to a higher incidence of autosomal recessive disorders [3,12]. The strong public awareness likely reflects the influence of national education campaigns and premarital screening efforts over the past twenty years. The study also revealed positive views on preventive genetic services, with over 90% supporting expanded premarital, prenatal, and newborn screening programs. These results align with earlier studies in the Western region and in Riyadh, where most people favoured genetic testing and supported government-led screening initiatives [10,13]. The broad acceptance of these programs suggests a growing public understanding of the importance of early diagnosis and disease prevention.

The current study shows public support for expanding premarital and newborn screening programs, indicating community recognition of their importance. However, while general knowledge is good, awareness of available genetic services remains limited; fewer than 40% of participants knew about specialized genetic labs or counselling clinics, and few had consulted a genetic counsellor. These findings are consistent with reports from Saudi Arabia and may also be relevant to other Middle Eastern populations that also have high rates of consanguineous marriage. The studies reported that public awareness of inherited disorders is good, but awareness of available genetic testing and counselling services remains limited [14,15]. This pattern indicates that increasing public awareness about genetic healthcare options, improving referral systems, and expanding access to specialised genetic counselling are ongoing public health priorities throughout the region. This gap between disease awareness and service knowledge reveals a critical shortfall in healthcare and education. This discrepancy highlights a key aspect of genetic literacy: recognizing genetic disease risk does not automatically mean understanding how to access or utilize genetic healthcare services. Improving genetic literacy should involve not only expanding knowledge about diseases but also enhancing public awareness of genetic testing, counselling, and screening options. The low use of genetic counselling services may be due to limited availability, lack of referral pathways, low public awareness, and the recent growth of genomic medicine in Saudi Arabia. Genetic counselling is vital for genetic disease prevention, risk assessment, reproductive choices, and psychosocial support [16]. Improved access could help close the gap between public understanding and clinical practice. Additionally, almost 48.2% of participants felt there was no improvement in genetic healthcare services, indicating that merely raising public awareness might not be enough. Future health promotion efforts should aim to enhance the visibility and accessibility of current genetic services via primary healthcare centres, community outreach initiatives, educational institutions, and digital platforms. These strategies could boost public trust in genetic counselling and testing, encouraging more appropriate use.

Multivariable logistic regression was conducted to identify independent factors associated with good awareness of genetic testing. Several demographic variables were evaluated; however, after adjusting for potential confounding variables, marital status remained the only independent predictor. Unmarried individuals had significantly higher odds of good awareness than married individuals. Although the reasons for this association cannot be determined from this cross-sectional study, these findings warrant further investigation.

Social media was identified as the primary source of genetic information, underscoring its role as a major channel for health communication and public education. While it offers opportunities to disseminate genetic knowledge, concerns about misinformation and the quality of online health content remain significant. Healthcare institutions and professional organisations could leverage these platforms to share accurate, evidence-based genetic information. The study’s findings also have notable implications for precision medicine and genomic healthcare in Saudi Arabia, where substantial investments have been made through initiatives such as the Saudi Human Genome Program. These efforts aim to enhance disease prevention, early diagnosis, and personalised healthcare through genomic technologies. The high acceptance of genetic testing and screening observed in this study may support the effective rollout of these initiatives at both the regional and national levels.

From a public health perspective, these results emphasise the need to enhance population-wide genetic services alongside public education. The strong acceptance of genetic screening programs offers an opportunity to broaden preventive genomics within primary healthcare, strengthen referral systems to specialised genetic services, and enable earlier identification of individuals and families at risk of inherited conditions. These initiatives could support informed reproductive choices, improve access to genetic counselling, and ultimately reduce the impact of preventable hereditary diseases. They also align with Saudi Arabia’s move towards precision medicine and genomic healthcare.

This study demonstrates several strengths. The sample size surpassed the minimum needed, allowing for an assessment of multiple aspects related to genetic diseases. Additionally, it examined disease-related knowledge, awareness, and familiarity with genetic healthcare services, offering insights into the difference between general awareness of genetic diseases and knowledge of available services. The study also supplies regional data on a relatively under-researched public health issue in Saudi Arabia.

One limitation to note is that, because the questionnaire was distributed via social media platforms, younger individuals, students, and those with higher digital literacy were more likely to participate. This is reflected in the study’s demographic profile, with most respondents being students and nearly half aged 21 to 25 years. The sample also had a higher proportion of female participants, which may reduce its representativeness. Consequently, the study sample may not fully represent the demographic characteristics of the wider Qassim population, particularly older adults and individuals with limited internet access or digital literacy. The higher proportion of younger participants and university students may have resulted in higher estimates of knowledge and awareness, as these groups generally have greater access to educational resources and health-related information. In addition, the findings were based on self-reported responses and may therefore be subject to recall and social desirability biases. Although the questionnaire underwent expert review for content validity, formal pilot testing and reliability assessment were not conducted. As a result, the internal consistency and reliability of the questionnaire could not be established. Therefore, the findings should be interpreted with caution and should not be considered fully generalisable to the entire regional population. Furthermore, because the study has a cross-sectional design, the observed associations should not be interpreted as evidence of causation.

Future research using larger, more representative samples should further investigate factors associated with public knowledge, awareness, and attitudes towards genetic diseases and genetic screening programs.

## 5. Conclusions

This study revealed positive attitudes towards genetic diseases and genetic screening among residents of the Qassim region. Participants demonstrated good recognition of the role of consanguinity in genetic diseases, strongly supported genetic screening programs, and expressed willingness to participate in genetic research. Nonetheless, awareness of and use of genetic services and counselling remain limited. These results reveal a significant gap between knowledge of the disease and awareness of accessible genetic healthcare services. These results emphasise the need for ongoing public education, expanded genetic counselling, and improved access to specialised genetic healthcare facilities. Improving regional genetic services and promoting evidence-based health education can support Saudi Arabia’s national goals for precision medicine and genomic healthcare. Public support for establishing a regional genetic laboratory reflects positive community views on expanding local genetic services. Additional research is needed to evaluate healthcare needs, feasibility, and the potential impacts of increasing regional genetic facilities, which will inform evidence-based healthcare planning in Saudi Arabia.

## Figures and Tables

**Table 1:** Sociodemographic characteristics of the participants.

Variable	Total N = 398 (%)	Male N = 112 (%)	Female N = 286 (%)	*p*-Value
Age group				<0.001
10–15	12 (3.0)	6 (5.4)	6 (2.1)	
16–20	66 (16.6)	5 (4.5)	61 (21.3)	
21–25	197 (49.5)	54 (48.2)	143 (50)	
26–30	52 (13.1)	23 (20.5)	29 (10.1)	
More than 36	71 (17.8)	24 (21.4)	47 (16.4)	
Educational level				0.001
Secondary school	79 (19.8)	18 (16.1)	61 (21.3)	
Bachelor	270 (67.8)	68 (60.7)	202 (70.6)	
Diploma	25 (6.3)	14 (12.5)	11 (3.8)	
Higher studies	9 (2.3)	5 (4.5)	4 (1.4)	
Other	15 (3.8)	7 (6.3)	8 (2.8)	
Marital status				0.168
Married	96 (24.1)	32 (28.6)	64 (22.4)	
Separated	8 (2.0)	-	8 (2.8)	
Single	292 (73.4)	79 (70.5)	213 (74.5)	
Widowed	2 (0.5)	1 (0.9)	1 (0.3)	
Occupation				<0.001
Student	328 (82.4)	93 (83.0)	235 (82.2)	
Retired	14 (3.5)	11 (9.8)	3 (1.0)	
Other	56 (14.1)	8 (7.1)	48 (16.8)	
Residence				0.960
Unaizah	98 (24.5)	27 (24.1)	71 (24.8)	
Bruiadah	127 (31.9)	37 (33.0)	90 (31.5)	
Al-Rass	28 (7.0)	6 (5.4)	22 (7.7)	
Al-Bukarya	61 (15.3)	17 (15.2)	44 (15.4)	
Al-Mznab	61 (15.3)	19 (17.0)	42 (14.7)	
Al-Badae	23 (5.8)	6 (5.4)	17 (5.9)	

**Table 2:** Participants’ knowledge and awareness of genetic diseases.

SN	Item	Responses				*p*-Value
			Total (%)	Male N = 112 (%)	Female N = 286 (%)	
1.	Do you know what the most common genetic diseases are in the Kingdom of Saudi Arabia?	Yes	264 (66.3)	76 (76.9)	188 (65.7)	0.687
		No	134 (33.7)	36 (32.1)	98 (34.3)	
2.	In your opinion, how prevalent are genetic diseases in the Qassim region?	Very common	183 (46.0)	47 (42.0)	136 (47.6)	0.272
		Not common	73 (18.3)	26 (23.2)	47 (16.4)	
		Don’t know	142 (35.7)	39 (34.8)	103 (36.0)	
3.	Do you believe that consanguineous marriage is one of the major causes of genetic diseases?	Yes	368 (92.5)	102 (91.1)	266 (93.0)	0.511
		No	30 (7.5)	10 (8.9)	20 (7.0)	
4.	Do you believe that genetic diseases are transmitted through infection?	Yes	25 (6.3)	7 (6.3)	18 (6.3)	0.987
		No	373 (93.7)	105 (93.8)	268 (93.7)	
5.	Do you know how you can reduce your risk of developing a genetic disease?	Yes	244 (61.3)	66 (58.9)	178 (62.2)	0.542
		No	154 (38.7)	46 (41.1)	108 (37.8)	
6.	Do you know how genetic diseases are diagnosed?	Yes	205 (51.5)	56 (50.0)	149 (52.1)	0.706
		No	193 (48.5)	56 (50.0)	137 (47.9)	
7.	In your opinion, can people diagnosed with genetic diseases have healthy children?	Yes	340 (85.4)	92 (82.1)	248 (86.7)	0.245
		No	58 (14.6)	20 (17.9)	38 (13.3)	

**Table 3:** Participants’ attitudes toward preventing genetic diseases.

SN	Item	Response				*p*-Value
			Total (%)	Male N = 112 (%)	Female N = 286 (%)	
1.	Is there anyone in your family born with a hereditary disease?	Yes	92 (23.1)	23 (20.5)	69 (24.1)	0.445
		No	306 (76.9)	89 (79.5)	217 (75.9)	
2.	Do you know where to go to find out if you have a genetic disease in your family?	Yes	234 (58.8)	63 (56.3)	171 (59.8)	0.519
		No	164 (41.2)	49 (43.8)	115 (40.2)	
3.	Do you know what you should do if you learn that you may pass on a genetic disease to your children?	Yes	226 (56.8)	48 (42.9)	124 (43.4)	0.928
		No	172 (43.2)	64 (57.1)	162 (56.6)	
4.	Do you support increasing the number of genetic diseases that must be screened for in premarital screening?	Yes	372 (93.5)	100 (89.3)	272 (95.1)	0.035
		No	26 (6.5)	12 (10.7)	14 (4.9)	
5.	Do you support increasing the number of genetic diseases that should be screened for in prenatal screening?	Yes	354 (88.9)	94 (83.9)	260 (90.9)	0.046
		No	44 (11.1)	18 (16.1)	26 (9.1)	
6.	Do you support increasing the number of genetic diseases that should be screened for in newborn screening?	Yes	361 (90.7)	95 (84.8)	266 (93.0)	0.011
		No	37 (9.3)	17 (15.2)	20 (7.0)	

**Table 4:** Participants’ awareness of health services for genetic diseases in the Qassim region.

SN	Item	Response				*p*-Value
			Total (%)	Male N = 112 (%)	Female N = 286 (%)	
1.	In your opinion, have health services provided to people with hereditary diseases been developed in the Qassim region?	Yes	206 (51.8)	49 (43.8)	157 (54.9)	0.045
		No	192 (48.2)	63 (56.3)	129 (45.1)	
2.	Are there laboratories specialized in genetic testing and genetic counselling clinics in your city?	Yes	154 (38.7)	42 (37.5)	112 (39.2)	0.760
		No	244 (61.3)	70 (62.5)	174 (60.8)	
3.	Do you support the decision to establish a regional laboratory to conduct genetic tests?	Agreed	374 (94.0)	103 (92.0)	271 (94.8)	0.280
		Don’t agree	3 (0.8)	2 (1.8)	1 (0.3)	
		I don’t know	21 (5.3)	7 (6.3)	14 (4.9)	
4.	Have you ever contacted a genetic counsellor?	Yes	18 (4.5)	5 (4.5)	13 (4.5)	0.972
		No	380 (95.5)	107 (95.5)	273 (95.5)	
5.	Have you ever participated in a study on genetic diseases?	Yes	42 (10.6)	9 (8.0)	33 (11.5)	0.306
		No	356 (89.4)	103 (92.0)	253 (88.5)	
6.	Are you ready to participate in studies on genetic diseases?	Yes	285 (71.6)	73 (65.2)	212 (74.1)	0.075
		No	113 (28.4)	39 (34.8)	74 (25.9)	

**Table 5:** Univariate and multivariable binary logistic regression analyses of variables associated with genetic testing awareness status among participants in the Qassim region.

Variable	Univariate Analysis		Multivariate Analysis	
	OR (95% CI)	*p*-Value	aOR (95% CI)	*p*-Value
Age	0.93 (0.80–1.09)	0.415	1.17 (0.92–1.48)	0.193
Sex	1.34 (0.86–2.09)	0.196	1.25 (0.79–1.98)	0.339
Education level	0.94 (0.74–1.21)	0.666	-	
Marital status				
Married	Reference		Reference	
Unmarried	1.73 (1.05–2.85)	0.029	2.34 (1.08–5.08)	0.030
Occupation	0.69 (0.51–0.94)	0.019	0.74 (0.54–1.02)	0.071
Residence	1.03 (0.93–1.15)	0.498	-	
Family history of hereditary diseases	1.02 (0.63–1.65)	0.922	-	

## Data Availability

The data presented in this study are available upon request from the corresponding author due to privacy/legal/ethical reasons.

## Supplementary Materials

The following supporting information can be downloaded at: https://www.mdpi.com/article/10.3390/healthcare14162549/s1.
