# Supplementary material for: Public Awareness of Genetic Diseases, Preventive Screening, and Genetic Healthcare Services in the Qassim Region, Saudi Arabia: A Cross-Sectional Study
Source: Healthcare (Basel). 2026 Aug 14;14(16):2549. doi: 10.3390/healthcare14162549 (PMC13512358; doi:10.3390/healthcare14162549)
Supplement: Supplementary file 1 [file healthcare-14-02549-s001.zip › healthcare-4449071-supplementary.pdf]

Supplementary Material (S1)

Questionnaire Used to Assess Public Awareness of Genetic Diseases, Preventive Screening, and Genetic Healthcare Services in the Qassim Region, Saudi Arabia

What is your age group?

☐ 10–15 years
☐ 16–20 years
☐ 21–25 years
☐ 26–30 years
☐ More than 36 years

What is your highest level of education?

☐ Secondary school
☐ Bachelor's degree
☐ Diploma
☐ Postgraduate studies
☐ Other

What is your marital status?

☐ Married
☐ Separated
☐ Single
☐ Widowed

What is your current occupation?

☐ Student
☐ Retired
☐ Other

Which city in the Qassim region do you currently reside in?

☐ Unaizah
☐ Buraidah
☐ Al-Rass

☐ Al-Bukayriyah
☐ Al-Mithnab
☐ Al-Badai

Do you know the most common genetic diseases in the Kingdom of Saudi Arabia?

☐ Yes
☐ No

In your opinion, how prevalent are genetic diseases in the Qassim region?

☐ Very common
☐ Not common
☐ Don't know

Do you believe that consanguineous marriage is one of the major causes of genetic diseases?

☐ Yes
☐ No

Do you believe that genetic diseases are transmitted through infection?

☐ Yes
☐ No

Do you know how you can reduce your risk of developing a genetic disease?

☐ Yes
☐ No

Do you know how genetic diseases are diagnosed?

☐ Yes
☐ No

In your opinion, can people diagnosed with genetic diseases have healthy children?

☐ Yes
☐ No

Has anyone in your family been diagnosed with a hereditary disease?

☐ Yes

☐ No

Do you know where to seek information if a genetic disease is present in your family?

☐ Yes

☐ No

Do you know what you should do if you learn that you may pass on a genetic disease to your children?

☐ Yes

☐ No

Do you support expanding the number of genetic diseases included in the premarital screening programme?

☐ Yes

☐ No

Do you support expanding the number of genetic diseases included in the prenatal screening programme?

☐ Yes

☐ No

Do you support expanding the number of genetic diseases included in the newborn screening programme?

☐ Yes

☐ No

In your opinion, have healthcare services for people with hereditary diseases improved in the Qassim region?

☐ Yes

☐ No

Are there specialised genetic testing laboratories and genetic counselling clinics in your city?

☐ Yes

☐ No

Do you support establishing a regional laboratory dedicated to genetic testing?

☐ Yes
☐ No
☐ I don't know

Have you ever consulted a genetic counsellor?

☐ Yes
☐ No

Have you ever participated in a research study on genetic diseases?

☐ Yes
☐ No

Would you be willing to participate in future research studies on genetic diseases?

☐ Yes
☐ No

What is your primary source of information about genetic diseases?

☐ Social media applications
☐ Internet websites
☐ Specialist physician
☐ Other

In your opinion, what are the main reasons for the spread of genetic diseases in the Qassim region? (Select all that apply.)

☐ Consanguineous marriage
☐ Lack of health awareness
☐ Genetic mutations
☐ Other

In your opinion, which of the following measures would be most effective in reducing the burden of genetic diseases in the Qassim region? (Select all that apply.)

☐ Raising health awareness
☐ Awareness campaigns
☐ Commitment to genetic screening results

☐ Other
